# Dependence of human cell survival and proliferation on the CASP3 prodomain

**DOI:** 10.1038/s41420-024-01826-6

**Published:** 2024-02-06

**Authors:** Ebrahim Eskandari, Gian Luca Negri, Susanna Tan, Margarita E. MacAldaz, Shengsen Ding, Justin Long, Karina Nielsen, Sandra E. Spencer, Gregg B. Morin, Connie J. Eaves

**Affiliations:** 1 Terry Fox Laboratory, British Columbia Cancer Research Institute, Vancouver, BC Canada; 2 Department of Medical Genetics, University of British Columbia, Vancouver, BC Canada; 3 Canada’s Michael Smith Genome Sciences Centre, BC Cancer Research Institute, University of British Columbia, Vancouver, BC Canada; 4 School of Biomedical Engineering, University of British Columbia, Vancouver, BC Canada

**Keywords:** Apoptosis, Cell growth

## Abstract

Mechanisms that regulate cell survival and proliferation are important for both the development and homeostasis of normal tissue, and as well as for the emergence and expansion of malignant cell populations. Caspase-3 (CASP3) has long been recognized for its proteolytic role in orchestrating cell death-initiated pathways and related processes; however, whether CASP3 has other functions in mammalian cells that do not depend on its known catalytic activity have remained unknown. To investigate this possibility, we examined the biological and molecular consequences of reducing CASP3 levels in normal and transformed human cells using lentiviral-mediated short hairpin-based knockdown experiments in combination with approaches designed to test the potential rescue capability of different components of the CASP3 protein. The results showed that a ≥50% reduction in CASP3 levels rapidly and consistently arrested cell cycle progression and survival in all cell types tested. Mass spectrometry-based proteomic analyses and more specific flow cytometric measurements strongly implicated CASP3 as playing an essential role in regulating intracellular protein aggregate clearance. Intriguingly, the rescue experiments utilizing different forms of the CASP3 protein showed its prosurvival function and effective removal of protein aggregates did not require its well-known catalytic capability, and pinpointed the N-terminal prodomain of CASP3 as the exclusive component needed in a diversity of human cell types. These findings identify a new mechanism that regulates human cell survival and proliferation and thus expands the complexity of how these processes can be controlled.

**Figure:**
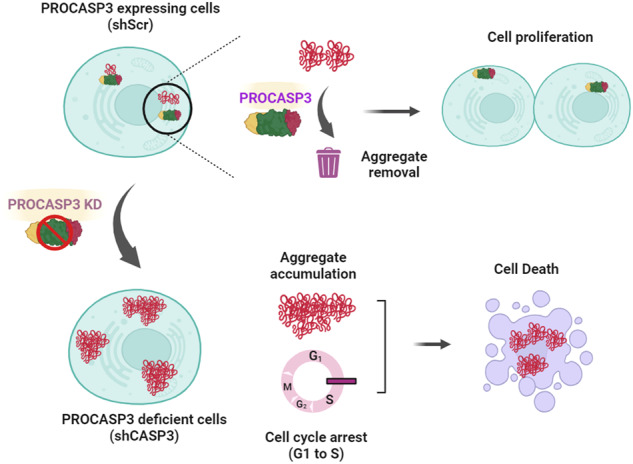
The graphical abstract illustrates the critical role of CASP3 for sustained proliferation and survival of human cells through the clearance of protein aggregates.

The graphical abstract illustrates the critical role of CASP3 for sustained proliferation and survival of human cells through the clearance of protein aggregates.

## Introduction

Caspase-3, also known as CASP3 in humans, belongs to a family of Cysteine-ASPartic proteASES (cysteine proteases). Initially, like related caspases, it is produced as an inactive zymogen. These are generically referred to as procaspases, whose latent proteolytic activity is then activated upon exposure to specific internal and/or external signals [1–3]. CASP3 is best known for its role in mediating the cleavage of specific proteins following the activation of apoptosis. The possibility that caspase-3 has other functions was first suggested from studies of model organisms, e.g., yeast [4], Drosophila [5, 6], and mice [7, 8] with selective deletion of caspase-3 gene sequences that include its conserved catalytic site [7–10]. These showed that, in mice, caspase-3-deficiency leads to abnormalities in osteogenesis [11] and cardiac [12] and skeletal muscle features [13], suggesting non-apoptotic roles, nevertheless shown to be mediated by caspase-3-dependent proteolytic degradation of intracellular proteins. [7–10]. However, if and how caspase 3 may have functional properties that are not dependent on its known catalytic domain has remained undefined.

These historic findings prompted us to investigate the potential range of activities of human CASP3 initially in normal and malignant human mammary cells as models, anticipating potential exploitable differences associated with their transformation. The findings led to the identification of multiple non-proteolytic biological activities (cell survival and proliferation) and biochemical activities (protein aggregate removal) of CASP3. This then prompted a further investigation of the molecular control of these activities of CASP3 and a demonstration of their generality to other human cell types.

## Results

### CASP3 is required for the expansion of normal and malignant human mammary cell populations

The mammary gland in normal adult human women consists of a two-cell layered tree-like structure with branching ducts that terminate in expanded lobules. The inner layer is composed of phenotypically distinct luminal progenitors (LPs) and luminal cells (LCs), and the outer basally positioned layer contains basal cells (BCs) [14]. LPs and BCs proliferate in vitro in the presence of EGF [14] and are thought to be the origin of most triple-negative and poorly treatable human breast cancers [15, 16].

**Fig. 1:**
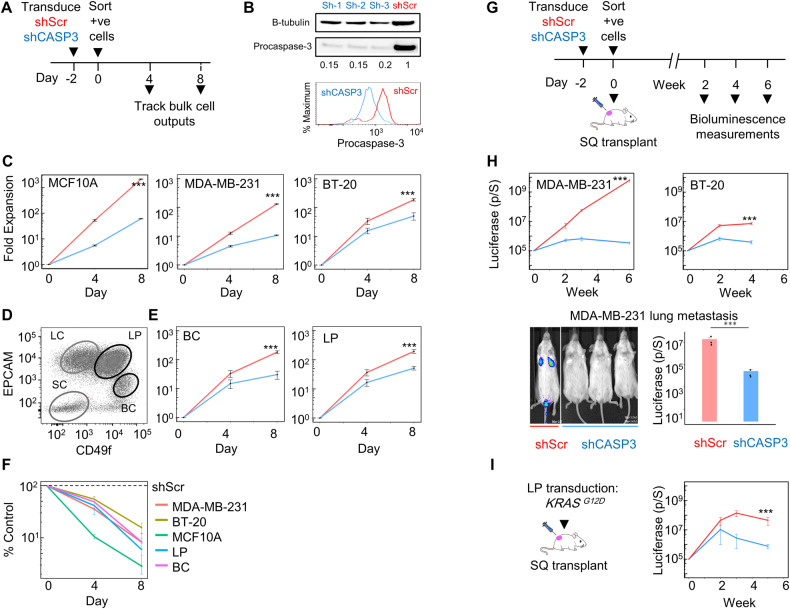
Requirement of CASP3 for normal and malignant human mammary cell population expansion. **A** Experimental plan to assess the effect of CASP3 KD on human mammary cell population expansion in vitro. Viable cell numbers were measured 4 and 8 days after sorting transduced cells. **B** WB and flow cytometric analysis of changes in PROCASPASE3 levels in shCASP3- or shScr-transduced MCF10A cells measured 2 days after sorting transduced cells (10^4^/cm^2^). **C** Reduced expansion of shCASP3- vs shSCR-transduced human mammary cells in 2D cultures. Data shown are the mean ± SEM of the Annexin V-PI- cell yields measured 4 and 8 days later in 3 experiments, each using a different CASP3 shRNA and the same shScr shRNA (***P < 0.001, Student’s *t*-test at day 8). **D** Representative FACS profile used to isolate normal human mammary BCs and LPs from viably cryopreserved pre-digested breast reduction samples. **E** Reduced expansion of BCs and LPs in 2D-cultures following CASP3-transduction (***P < 0.001, Student’s *t*-test of day 8 data from 3 experiments each with different donor cells). **F** Comparison of the differential effects of CASP3 KD on the different cell types analyzed. Data shown are the % yields of viable CASP3-transduced cells relative to their SCR-transduced-controls (all differences compared to controls are significant, *P* < 0.001, Student’s *t*-test) **G** Experimental plan of the in vivo experiments. **H** Bioluminescence measurements used to track tumor growth over time in groups of 3 immunodeficient adult female non-obese diabetic-Rag1-/--IL2Rγ-/- (NRG) mice transplanted with 10^3^ luciferase-transduced MDA-MB-2^3^1 or 10^4^ luciferase-transduced BT-20 cells either SQ (upper panels), or 10^3^ luciferase-transduced MDA-MB-2^3^1 cells injected IV and examined 1 week later (lower panels). Shown in each case are the mean ± SEM of values from 3 separate experiments, each using a different one of the 3 *CASP3* shRNAs and the same shScr shRNA (***P < 0.001, Student’s *t*-test). Ell (**I**) Bioluminescence measurements used to track tumor growth over time in groups of 3 NRG mice, each transplanted SQ with 10^4^ (sorted) *KRAS*^*G12D*^ -transduced LPs (isolated from a different donor in each of 3 experiments) and co-transduced with a luciferase vector (***P < 0.001, Student’s *t*-test).

To investigate the potential ability of CASP3 expression to modulate the growth properties of human mammary cells, we employed a knockdown (KD) strategy using lentiviral short hairpin (sh) vectors targeting CASP3, along with scrambled (Scr) controls (Fig. 1A). Western blot (WB) and flow cytometric analyses were initially performed on a triple negative breast cancer (TNBC) cell line MDA-MB-231 [17], and an immortalized but non-tumorigenic MCF10A [18] mammary epithelial cell line over 2 days following their transduction. The KD consistently produced a significant 5–7-fold reduction in PROCASPASE-3 levels in the shCASP3-treated cells compared to controls (Fig. 1B and S1A, B). Examination of the impact of CASP3 KD on the in vitro expansion of the MCF10A and MDA-MB-231 cell lines, and BT-20 [19], another TNBC cell line, consistently demonstrated a significant albeit variable decrease in the output of the shCASP3 cells compared to their shScr-transduced controls (Fig. 1C). These results provided the first indication that maintenance of physiological levels of CASP3 expression are required to support the proliferation of both normal and malignant human mammary cells in vitro. Examination of the impact of CASP3 KD on FACS-purified normal EGF-responsive human mammary BCs and LPs also showed a significant reduction in their clonogenic output of these primary cells (Fig. 1D–F).

We then asked whether this effect of CASP3 KD would affect the in vivo tumorigenic potential of transformed human mammary cells. Notably, by comparison to the shScr-transduced controls, CASP3 KD consistently suppressed the growth of tumors from both of these cell types (Fig. 1G, H). This effect of CASP3 KD was also found to extend to the lung colonizing ability of intravenously (IV) injected MDA-MB-231 cells (Fig. 1H, lower panel). Similarly, CASP3 KD reduced the growth of the slow-growing tumors normally generated from *KRAS*^*G12D*^-transduced human mammary LPs (Fig. 1I) [20]. These findings support the conclusion that CASP3 expression is required for the in vivo expansion of nascent as well as established malignant human mammary cells.

### CASP3 is required for cell cycle progression from G0/G1 to S

**Fig. 2:**
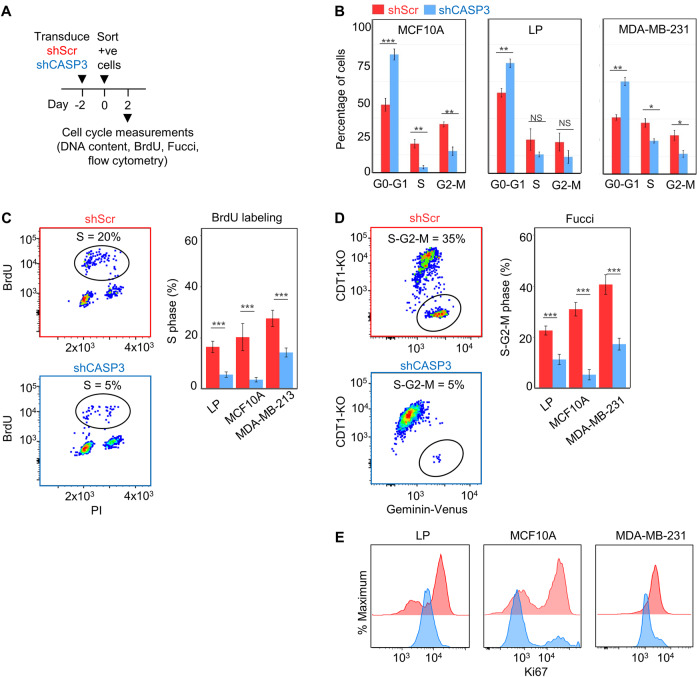
ShCASP3-transduced cells show a rapid blockade of cell cycle progression at the G0-G1 to S transition. **A** Experimental plan for analysis of effects of shCASP3 on cell cycling. **B** FACS analysis of the DNA content of PI-stained shCASP3- and shScr-transduced LPs, MCF10A and MDA-MB-231 cells 2 days after their isolation by FACS as indicated in panel A (***P < 0.001, **P < 0.01, Chi-square test). **C** Results of FACS analysis of BrdU labeling of shCASP3- and shScr-transduced cells 2 days after their isolation by FACS and following a 2-h exposure to BrdU. Shown are the mean ± SEM of 3 technical replicates for each of the 3 different cell types analyzed. **D** Results of FACS analysis of Fucci-transduced cloned isolates of the 3 cell types. **E** Comparative FACS analysis of antibody-stained intracellular Ki67 in shCASP3- and shScr-transduced cells.

We then designed a series of time course experiments to determine whether the observed pervasive inhibitory effect of CASP3 KD on cell expansion might be due to an effect on cell division control (Fig. 2A). Initial measurements of propidium iodide (PI) staining (Fig. 2B) showed the percentage of viable cells (≥2n) in G0-G1 in bulk asynchronously expanding LPs, MCF10A, and MDA-MB-231 cell populations was consistently higher in the shCASP3-transduced cells compared to the shScr controls (Fig. 2B). In contrast, the corresponding percentage of viable cells in S and G2/M was lower in the shCASP3-transduced cells compared to shScr controls. These findings were supported by the results of BrdU-labeling experiments that showed the percentage of the same sources of cells progressing into S-phase to be consistently significantly lower in the shCASP3-transduced group compared to the matched shScr controls (Fig. 2C).

For more definitive analyses, we generated Fluorescent Ubiquitination-based Cell Cycle Indicator (Fucci)-labeled MCF10A and MDA-MB-231 cells [21]. After 3 weeks of expansion of FACS-isolated single Fucci cells selected clones transduced with either shCASP3 or shScr constructs were analyzed by FACS (Fig. S1D). The results again showed a significantly reduced percentage of shCASP3-transduced cells in the S-G2-M phase after 2 days (Fig. 2D).

Additional molecular comparison of the effect of CASP3 KD cells was undertaken using intracellular flow cytometry to compare the levels of expression of Ki67 (Fig. 2E), pRB1, CDK3, CDK4, CDK6, and cyclins C/D in the shCASP3 and shScr-transduced cells (Fig. S1F). The results confirmed that most of these proteins were lower in the shCASP3-transduced cells compared to the shScr controls. Nevertheless, forced expression of either CDK4 or 6, or Cyclin D did not rescue the proliferative capacity of the shCASP3 cells (Fig. S1G).

### CASP3 is required for cell survival

**Fig. 3:**
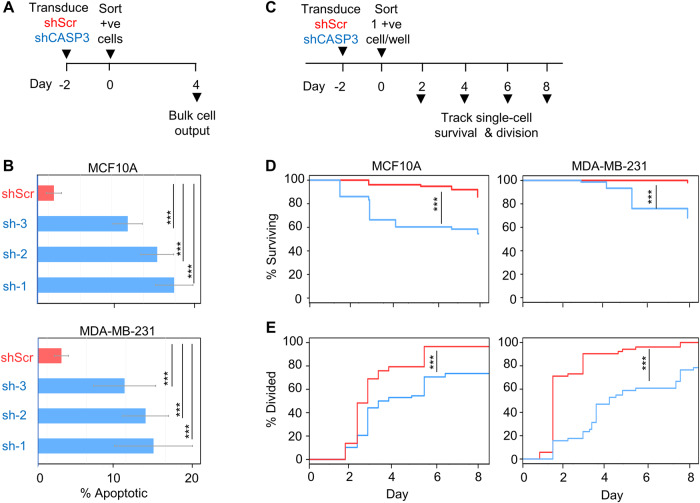
ShCASP3 induces rapid cell death. **A** Experimental plan for tracking the loss of viability in shCASP3- and shScr-transduced cells in bulk cultures. **B** Flow cytometrically determined proportions of Annexin V+ and/or PI + MCF10A and MDA-MB-231 cells assessed 3 days after FACS isolation of CASP3 shRNAs or shScr transduced cells. Values shown are the mean ± SEM of data from 3 separate experiments, each using CASP3 shRNA#1 and the same shScr shRNA (***P < 0.001, Student’s *t*-test). **C** Experimental plan for assessing the proliferation and death of single cells in culture. **D** Kaplan–Meier (KM) survival plots showing rapidly induced death in individually tracked shCASP3-transduced cells. Data are from 72 wells each initially containing a single shCASP3-transduced cell and 72 wells similarly initiated with a single shScr-transduced cell. Results have been compared using a log-rank test (***P < 0.001). **E** Cumulative plots of the timing of the first division (≥2 cells/well) of shCASP3- and shScr-transduced cells plated in (**D**) and compared using the Komoglerov–Smirnov test (***P < 0.001).

We next designed experiments to determine whether CASP3 might also have a key role in regulating normal and/or malignant human mammary cell survival. For these, we first used flow cytometry to examine bulk cultures of shCASP3- and shScr-transduced MCF10A and MDA-MB-231 cells stained with Annexin V and PI, 3 days post-transduction (Fig. 3A). These analyses showed a higher percentage of apoptotic cells in the shCASP3-transduced group compared to the shScr groups of both cell types analyzed (Fig. 3B). To investigate the timing of the effects of reduced CASP3 expression on cell survival, individual transduced cells from the shCASP3 and shScr groups were sorted into separate wells of 96-well plates and the single-cell cultures then monitored visually using a microscope to assess their presence and numbers (Fig. 3C). Cells were considered viable if their morphology resembled the refractile epithelial cells seen in concurrently plated bulk cultures. However, in the single-cell cultures, dead cells were identified by their loss of refractility and inability to adhere to the well surface. Decreased survival of shCASP3-treated cells was more rapidly evident and appeared more pronounced in the single-cell cultures of the MCF10A cells compared MDA-MB-231 cells (Fig. 3D), consistent with the results obtained in the bulk culture experiments (Fig. 3A). Of additional interest was the observation that a significant proportion of the MCF10A and MDA-MB-231 cells subjected to CASP3 KD were still capable of undergoing at least one division after 60 h, albeit at a considerably slower rate compared to the shScr-transduced cells (i.e., <20% of the shCASP3-transduced MCF10A cells compared to >40% of the shScr-transduced controls (Fig. 3E and Fig. S1C).

### Global proteome analyses point to CASP3 as a regulator of protein aggregate accumulation

**Fig. 4:**
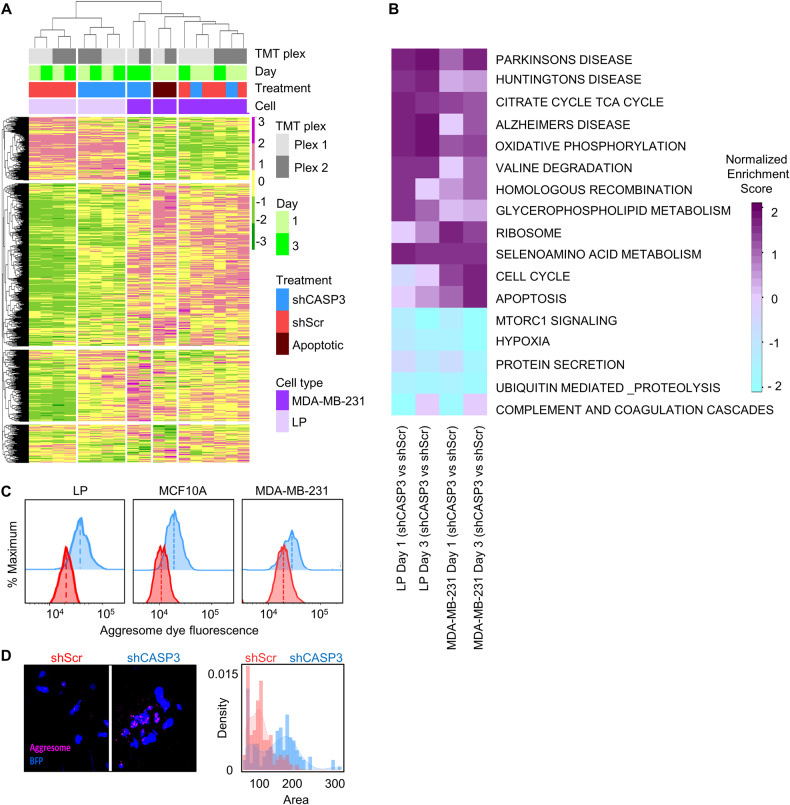
shCASP3-transduced cells contain rapidly increased levels of protein aggregates. **A** Global proteome analysis of shCASP3- and Scr-transduced LPs and MDA-MB-231 cells analyzed 1 and 3 days after their FACS isolation 2 days following lentiviral vector exposure. The combined heatmap and dendrogram shows the protein variance where the columns indicate the samples analyzed and the rows indicate individual proteins identified (total = 5211). Color key indicates Z-scores. **B** GSEA showing selectively altered pathways in shCASP3-transduced compared to Scr-transduced LPs and MDA-MB-231 cells on days 1 and 3. **C** Flow cytometric comparison of protein aggregate levels in shCASP3 and shScr–transduced cells showing these are consistently higher in the shCASP3-transduced cells (10^5^ cells/group/cell type). **D** Increased protein aggregate accumulation in shCASP3-transduced MCF10A cells. Representative immunofluorescent imaging (left panels) and quantification of aggregate levels/cell in shCASP3 and shScr–transduced cells.

To gain molecular insight into the mechanisms by which CASP3 mediates its pro-survival and pro-cell proliferation control, we designed a pilot mass spectrometry (MS) experiment to compare the global proteome changes in shCASP3- versus shScr-transduced normal primary human LP cells from a pool of 5 donors and malignant MDA-MB-231 mammary cells. The transduced cells were isolated by FACS 1 and 3 days after our standard 2-day transduction/expression protocol and cell lysates analyzed by tandem mass tag (TMT)-based MS [22]. As a positive control for apoptotic cells, MDA-MB-231 cells were treated with staurosporin for 18 h, then washed and processed in parallel (Fig. S2A). These experiments identified a total of 5941 proteins that were expressed in at least one of the samples, of which 5211 were expressed in all samples. Heatmaps and dendrograms comparing the levels of these revealed how the expression of the proteins clustered between the two treatments (Fig. 4A). Interestingly, these results suggest that the proteome of shCASP3-transduced LPs remained more similar to their controls than to shCASP3-transduced MDA-MB-231 cells, as shown in the correlation plots in Fig. S2B, despite their similarly reduced levels of CASP3 (Fig. S1B) and decreased proliferative activity following CASP3 KD (Fig. 1C).

Gene set enrichment analysis (GSEA) performed on the data for both cell types suggested upregulation of pathways involved in protein aggregation indicated by terms related to neurodegenerative diseases (Parkinson, Huntington, and Alzheimer disease), oxidative phosphorylation, and apoptosis in shCASP3 cells in both normal LPs and malignant MDA-MB-231 cells (Fig. 4B). Implicated downregulated pathways in shCASP3 cells were mTOR signaling and ubiquitin-mediated proteolysis. These findings point to a rapid effect of CASP3 KD on mechanisms that normally regulate abnormal protein accumulation.

### shCASP3-transduced cells contain increased levels of protein aggregates

We then used an aggresome detection kit [23–25] to compare the intracellular levels of misfolded protein aggregates (Fig S3A–C). Flow cytometric analysis indicated consistently higher protein aggregates in the shCASP3 cells compared to the control cells (Fig. 4C) and this was further supported by immunofluorescent (IF) imaging of shCASP3 vs shScr cells (Fig. 4D). The proteome data also showed higher levels of some proteins involved in autophagy (i.e., WIPI1) and ER stress (i.e., ATF6, HSPs, and DNAJB chaperons) in the shCASP3-transduced cells (Fig. S3D), suggesting an interplay between aggresome formation, autophagy, and ER stress response to maintain cellular homeostasis and protein quality control. However, an elevation of these proteins in shCASP3 cells might be a compensatory mechanism to mitigate enhanced levels of protein aggregates in these cells.

### The pro-survival function of CASP3 in human mammary cells is mediated by its prodomain

**Fig. 5:**
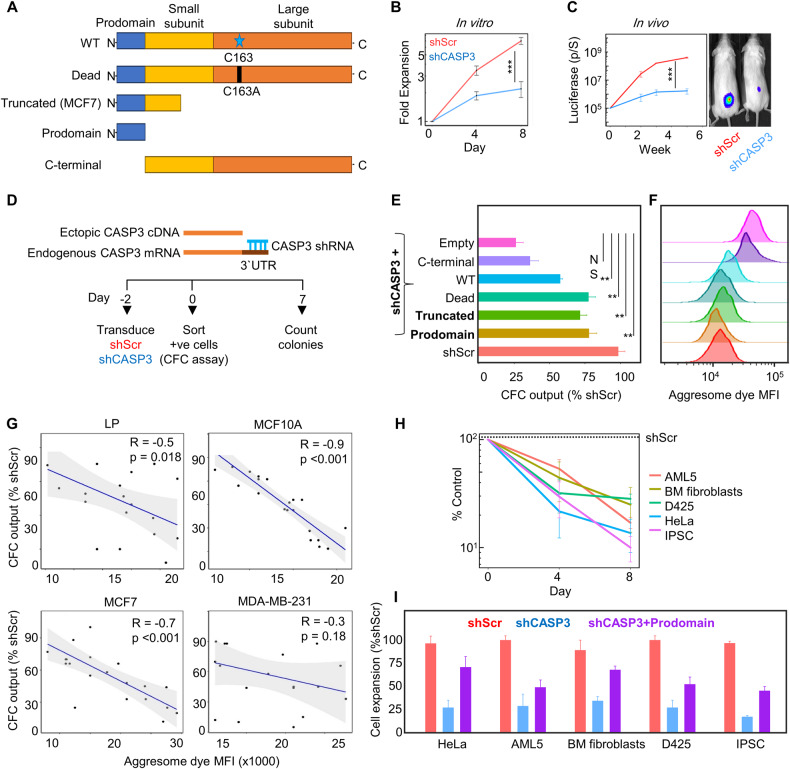
Rescue experiments demonstrate a pro-survival function of the CASP3 prodomain. **A** Schematic representation of CASP3 cDNAs used for the rescue experiments. **B** Reduced expansion in vitro of shCASP3-transduced MCF7 cells as compared to shScr–transduced control cells. Shown are the mean ± SEM of the results from 3 replicate experiments. **C** Changes in bioluminescence measurements used to track tumor growth in groups of 3 NRG mice, transplanted SQ with 10^4^ MCF7 cells transduced with vectors encoding Luc-YFP and either shCASP3-GFP or shScr-GFP (***P < 0.001, Student’s *t*-test at the last time point). **D** Outline of rescue experiments designed to test the ability of vectors that express different forms of CASP3 (shown in **A**) to restore the ability of shCASP3-transduced cells to produce colonies after 7 days. **E** Different CASP3 cDNAs transduced into simultaneously shCASP3-transduced MCF7 cells partially restores clonogenic activity. Shown are the mean ± SEM of 3 biological replicates (** indicates *P* < 0.01, NS = *P* ≥ 0.05. **F** Representative histograms of protein aggregate profiles measured by flow cytometry in matching cells in Panel E (10^5^ cells/group/cell type). **G** Correlation plots of protein aggregate levels determined as in (**F**) vs the CFC output of shCASP3-transduced cells expressing the CASP3 N-terminal prodomain. **H** The yields of viable cells after 4 and 8 days in cell type-specific growth-promoting cultures initiated with FACS-sorted transduced cells were assessed and compared. Expansion of shCASP3**-**transduced non-mammary cell types is consistently significantly reduced (all values vs shScr are *P* < 0.001; Student’s *t*-test at the last time point). **I** Overexpression of the CASP3 prodomain partially restores the expansion of shCASP3-transduced non-mammary cell types (all values vs shCASP3 are *P* < 0.001).

To determine how the CASP3 protein mediates its pro-survival function at a molecular level, we first examined the biological properties of MCF7 breast cancer cells, which expresses a truncated version of the normal human *CASP3* gene. Specifically, MCF7 cells have a 47-bp deletion in exon-3 of the *CASP3* gene that causes a translation stop prior to the proteolytic domain underlying their deficient protease activity (Fig. 5A) [26, 27], that we confirmed by PCR amplification (Fig. S4A, B). We asked what effect transduction of MCF7 cells with *CASP3* shRNAs that target the 3′-UTR of the *CASP3* transcript would have on their viability. This treatment reduced *CASP3* mRNA levels 3-fold as measured by qPCR (Fig S4C–E). Notably, the in vitro expansion of shCASP3-transduced MCF7 was significantly less than that obtained in the shScr cells (Fig. 5B). Likewise, CASP3 KD resulted in a notable reduction in the rate of MCF7 tumor growth in vivo (Fig. 5C). These findings highlight the presence of a preserved pro-survival role within the truncated, proteolytically inactive form of CASP3 produced in MCF7 cells, suggesting its potential relevance to the effects and mechanisms identified in cells with a complete CASP3 gene.

To formally test this idea, we undertook a series of experiments designed to test the ability of different modified CASP3 cDNA sequences (Fig. 5A, Fig. S4C–E) to rescue the impaired growth of shCASP3-transduced MCF7 cells. For this, shRNAs that target the 3′-UTR of the endogenous *CASP3* gene, including the MCF7 *CASP3* mRNA, were used so that they would not recognize the exogenously introduced active and inactive forms of CASP3 constructs (Fig. 5D). The CASP3 overexpressing vectors were validated by WB analysis, intracellular flow cytometry, and qPCR demonstrating that the shCASP3-transduced cells expressing either the proteolytically active or inactive CASP3 constructs contained similar levels of PROCASPASE-3 protein as the shScr group (Fig. S4C–E).

We then designed a series of experiments to localize more precisely, the functional portion of the CASP3 protein required for MCF7 cell survival and proliferation. These involved transducing MCF7 cells with the following types of constructs: (i) one shRNA targeting the endogenous 3′-UTR of the *CASP3* gene, along with (ii) one containing a full-length CASP3 cDNA, or (iii) one containing a full-length CASP3 cDNA but with a C163A mutation to render it proteolytically inactive, (iv) one containing a truncated CASP3 cDNA expressed in MCF7 cells, (v) one containing a CASP3 cDNA containing just the N-terminal prodomain, (vi) one containing just the C-terminal construct lacking the prodomain, and (vii) one consisting of an empty vector as a control. MCF7 cells were transduced with one of each of these and transduced (mCherry+YFP+) cells were isolated by FACS 2 days later and seeded in culture plates at a low density (Fig. 5D). The number of colonies counted a week later showed the different forms of CASP3 containing the N-terminal prodomain were able to significantly restore the impaired growth of shCASP3 MCF7 cells both in vitro (Fig. 5E) and in vivo (Fig. S4F). However, most importantly, the ectopic expression of the C-terminal fragment of CASP3 lacking the N-terminal prodomain failed to restore the impaired growth of shCASP3 MCF7 cells.

Extension of this same experimental strategy to a variety of normal and malignant human mammary cells with full-length *CASP3* gene (i.e., LPs, MCF10A, and MDA-MB-231 cells) showed a similar reversal of the shCASP3-mediated suppression of cell expansion in vitro when transduced with the same *CASP3* constructs and to the same extent obtained with a wild-type PROCASPASE-3 vector (Fig. S5A–F). Interestingly, the overexpression of different forms of CASP3 containing the N-terminal prodomain in shCASP3 cells resulted in reduced levels of protein aggregates to a similar level seen in shScr- transduced cells (Fig. 5F). However, shCASP3 cells overexpressing the C-terminal fragment and lacking the prodomain retained high levels of protein aggregates to the same extent as shCASP3 cells. A negative correlation was also found between the aggresome dye fluorescent intensity and the Colony forming cell (CFC) output of shCASP3-transduced cells expressing CASP3 N-terminal prodomain (Fig. 5G).

These findings indicate that the control of normal and malignant human mammary cell survival and proliferation is mediated exclusively and specifically by the prodomain of CASP3, independent of its known catalytic activity, and that this function may involve regulating the removal of protein aggregates.

### The CASP3 prodomain has a generically conserved requisite pro-survival function

We asked whether this pro-survival function of the CASP3 N-terminal prodomain might, like the conserved role of its catalytic domain in mediating apoptosis, extend to other cells types. For these pilot experiments, we selected a variety of normal (primary) and malignant human cells of different tissue origins; i.e., cells from the human embryonic fibroblast-derived-induced pluripotent stem cell (iPSC) line [28]; established cultures of normal human bone marrow (BM)-derived fibroblasts [29], cells from an acute myeloid leukemia cell line (AML5) [30], cells from a cervical cancer cell line (HeLa) [31], and cells from a medulloblastoma (D425) [32] cell line. The yields of viable cells after 4 and 8 days in cell type-specific growth-promoting cultures initiated with FACS-sorted transduced cells demonstrated that all tested cell types showed significantly reduced growth when transduced with shCASP3 (Fig. 5H), thus establishing a dependence on a certain level of continued CASP3 expression. Moreover, and importantly, introduction of the CASP3 N-terminal prodomain again led to a restoration of cell proliferation across all of these cell types (Fig. 5I).

These findings establish the generality of the required CASP3 prodomain-mediated pro-survival function in human cells.

## Discussion

The key finding from this study is that both normal and malignant human cells of multiple types, and likely all cell types, given their validation in human iPSCs, rely on expression of the CASP3 prodomain for their survival and proliferation. Initially, this finding was developed from a detailed examination of the responses of a variety of normal and malignant human mammary cells. However, the failure to identify any human mammary cells whose viability was not sensitive to CASP3 KD when assayed either in vitro or in vivo in transplanted immunodeficient mice suggested a potentially more preserved generic function of CASP3, which experiments with a variety of human cell types confirmed. The fact that our CASP3 KD and prodomain rescue experiments in every cell type tested also showed an exclusive and specific pro-survival function of the N-terminal prodomain reinforces the concept that this pro-survival function of CASP3 is likely an evolutionarily conserved component and activity of CASP3 (Fig S5G). Until recently, most non-apoptotic functions of caspase-3 were believed to be linked to its known proteolytic activity, allowing CASP3 to cleave various intracellular proteins to impact signaling pathways and thereby altering intrinsic or neighboring cell behavior [1, 33]. A comparable catalytic-independent role of Procaspase-3 has been documented in mouse embryonic fibroblasts (MEFs), where caspase-3 was found to influence fibronectin secretion, cell morphology, adhesion, and migration independent of cell cycle changes. This study suggested an involvement of ER–Golgi transport or vesicle trafficking, but the precise mechanisms by which procaspase-3 regulates these properties remained unexplored [34].

The second significant finding from this work is the evidence that the pro-survival role of the CASP3 prodomain appears to act by regulating protein aggregate accumulation possibly via a CASP3-dependent activation of autophagy. Our first results suggesting this mechanism came from comparative global proteome analyses of CASP3 KD and intact human mammary cells. Additional evidence from intracellular FACS analyses of protein aggregate accumulation provided more extensive documentation of the correlation of selective presence of an intact CASP3 prodomain and decreased aggregate levels.

While considerable research has focused on understanding the functions o the large (p20) and small (p10) subunits of procaspase-3 that contain its well-recognized proteolytically active site, the functional properties of the CASP3 N-terminal prodomain have remained largely unexplored. However, a suggested regulatory role of the caspase-3 prodomain in the context of mouse embryonic fibroblast apoptosis control was reported [35]. In that study, it was shown that a region within caspase-3 prodomain negatively regulates the activation of caspase-3 and its cleavage by caspase-9 is required for complete activation of caspase-3. This provides additional support for the concept of an evolutionary conservation of caspase-3 prodomain to regulate caspase-3 activation and induction of apoptosis [35]. Our findings now add another rationale for the evolutionary conservation of CASP3 prodomain in relation to its pro-survival function during evolution.

Protein aggregates are known for their toxic impact on cells, disrupting crucial cellular processes by interacting with cellular membranes or sequestering essential protein complexes [36, 37]. The accumulation of protein aggregates observed here in shCASP3-transduced cells is consistent with this result causing the observed initial delay in cell cycle progression, and decreasing cell viability and cell proliferation. Remarkably, the forced overexpression of catalytically active, inactive, or just the N-terminal prodomain of CASP3 were all found to reduce the otherwise increased levels of protein aggregates. This suggests a previously unknown mechanism for clearing protein aggregates from cells requiring the CASP3 N-terminal prodomain.

It is interesting to note how these findings build on previous evidence from studies of the role of ScMCA1 in yeast. ScMCA1 is the forerunner of CASP3 in yeast and ScMCA1-deficient yeast cells also accumulate misfolded protein aggregates [38, 39], leading to delays in cell cycle progression [40, 41]. This role in removing protein aggregates in yeast involves both the catalytic and non-catalytic functions of ScMCA1 [38, 39]. However, the specific part of ScMCA1 responsible for this action has remained uncertain, as has the mechanisms through which ScMCA1 reduces protein aggregates independently of its catalytic activity. ScMCA1 may have a chaperone-like function through its Q/N rich prodomain, thereby aiding in the reduction of misfolded aggregates within cells and a similar mechanism may have been preserved through evolution in cells of many organisms including humans. Interestingly, a recent study has suggested that CASP3 may exert both catalytically dependent and independent effects on proteostasis [42]. It revealed that CASP3 can induce disaggregation of TDP43 independent of cell cycle kinetics, ultimately promoting normal mitochondrial function in skeletal muscle myocytes.

Apoptosis and proteostasis also exhibit considerable crosstalk, as a number of recent studies have identified multifunctional proteins that mediate the two cellular functions in evolutionarily distant species [43, 44]. For example, Mca1 and Mca2 have been reported to regulate stress responses in *Magnaporthe oryzae* by promoting the clearance of insoluble aggregates [44]. Similar reports suggest that *Ustilago maydis* Mca1 N-terminal subunit plays a role in the proteostasis of this organism by inducing the clearance of stress-induced intracellular insoluble protein aggregates by directing Mca1 localization to protein aggregates [45]. These results add further support to the concept that the CASP3 N-terminal prodomain functions as a regulator of protein aggregate accumulation. In addition to the enrichment of pathways related to protein aggregation, our proteome data showed a possible effect of CASP3 KD on autophagy and ER stress as shown by changes in the levels of proteins involved in these pathways (i.e., ATF6, HSPs proteins). Future studies will clearly be of interest to elucidate how CASP3 may be involved in regulating cell behavior via effects on autophagy and the precise mechanism(s) of cell death involved.

In summary, this study reveals a consistent, albeit variably penetrant dependence of all cell types tested on the CASP3 prodomain for maintaining their survival, proliferation, and cell cycle progression. Strong evidence is also presented to suggest this function involves an essential role in the normal clearance of protein aggregates. This novel role appears to reflect a previously unknown but evolutionarily conserved role exhibited by its homolog, ScMCA1, in yeast.

### Supplementary information

S1

S2

S3

S4

S5

Original Data File

Supplemental Material

## Data Availability

The mass spectrometry proteomics data have been deposited to the ProteomeXchange Consortium (https://proteomecentral.proteomexchange.org/) via the PRIDE partner repository with the dataset identifier PXD045234.
