# Supplementary figures and images for: Dependence of human cell survival and proliferation on the CASP3 prodomain
Source: Cell Death Discov. 2024 Feb 6;10:63. doi: 10.1038/s41420-024-01826-6 (PMC10847432; doi:10.1038/s41420-024-01826-6)

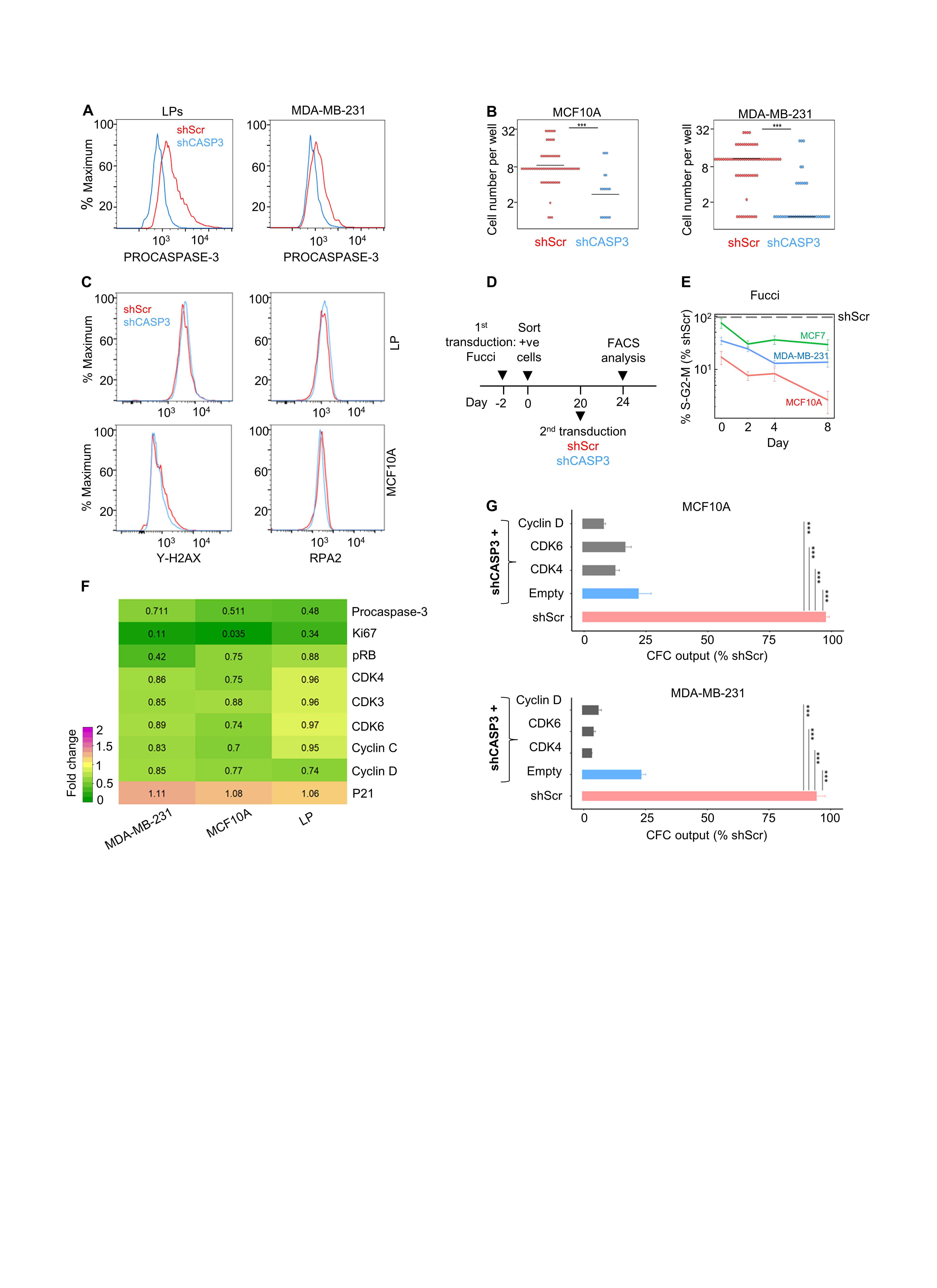

Supplement: Supplementary file 1 — S1 [file 41420_2024_1826_MOESM1_ESM.png]

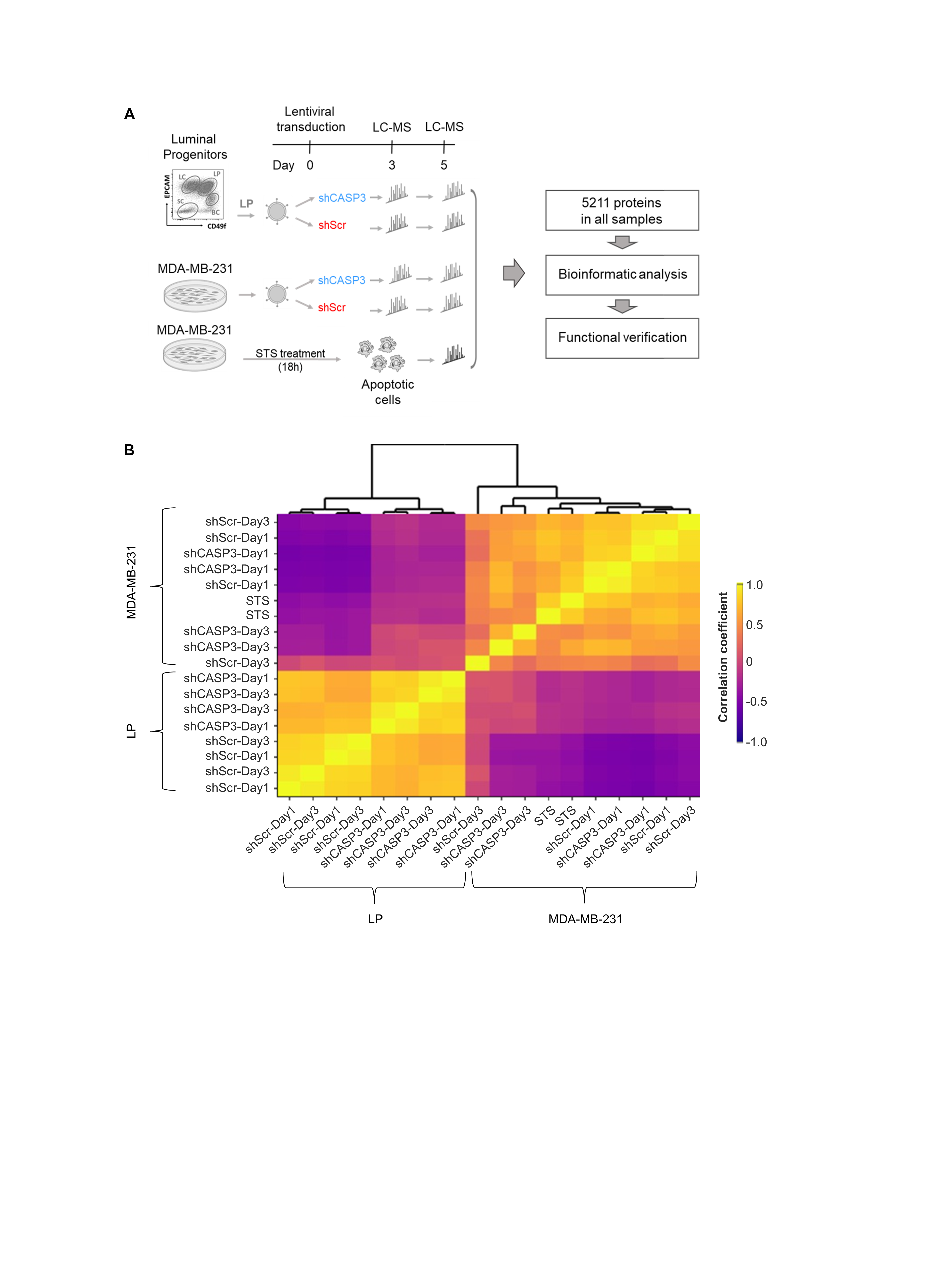

Supplement: Supplementary file 2 — S2 [file 41420_2024_1826_MOESM2_ESM.png]

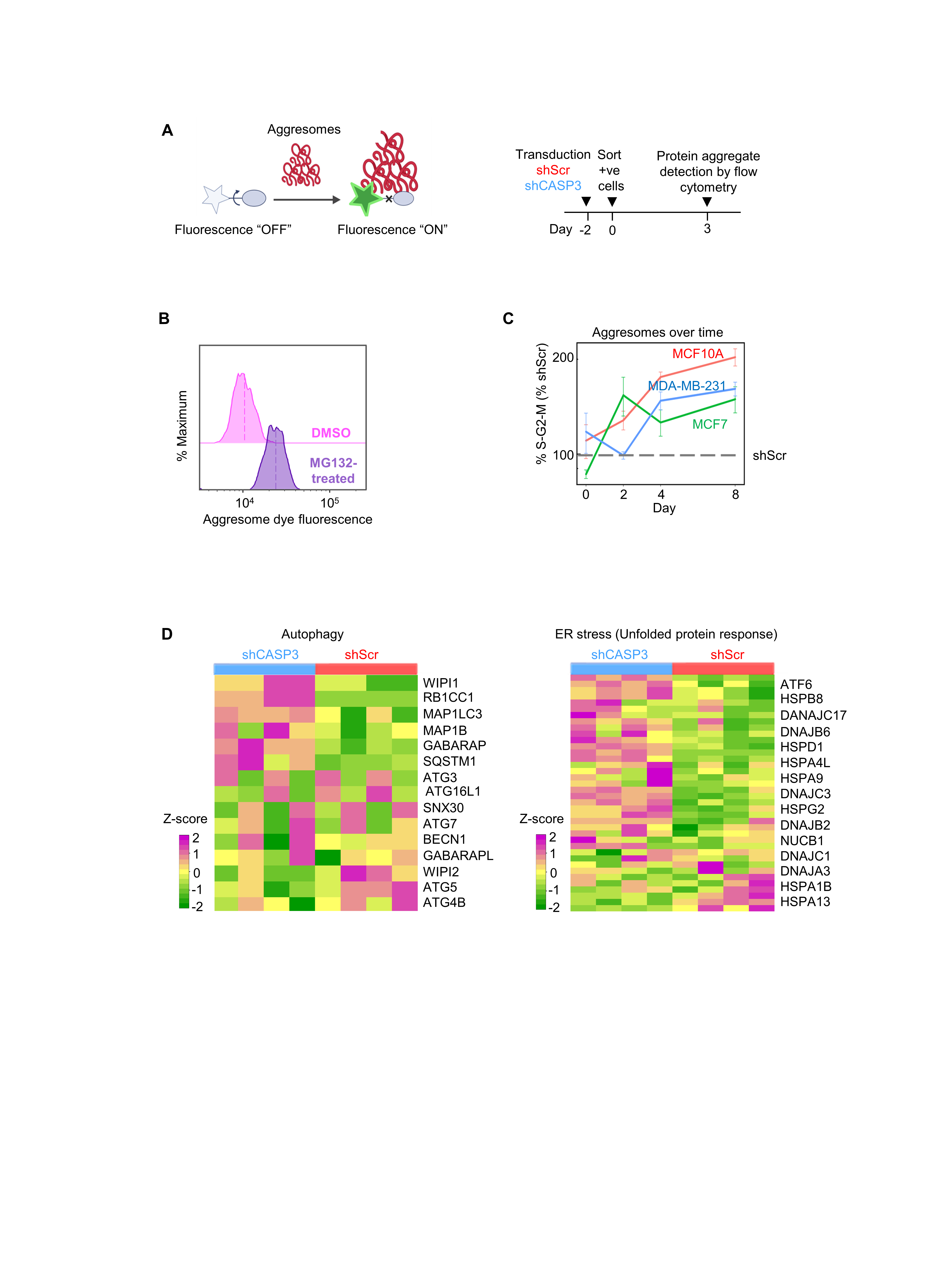

Supplement: Supplementary file 3 — S3 [file 41420_2024_1826_MOESM3_ESM.png]

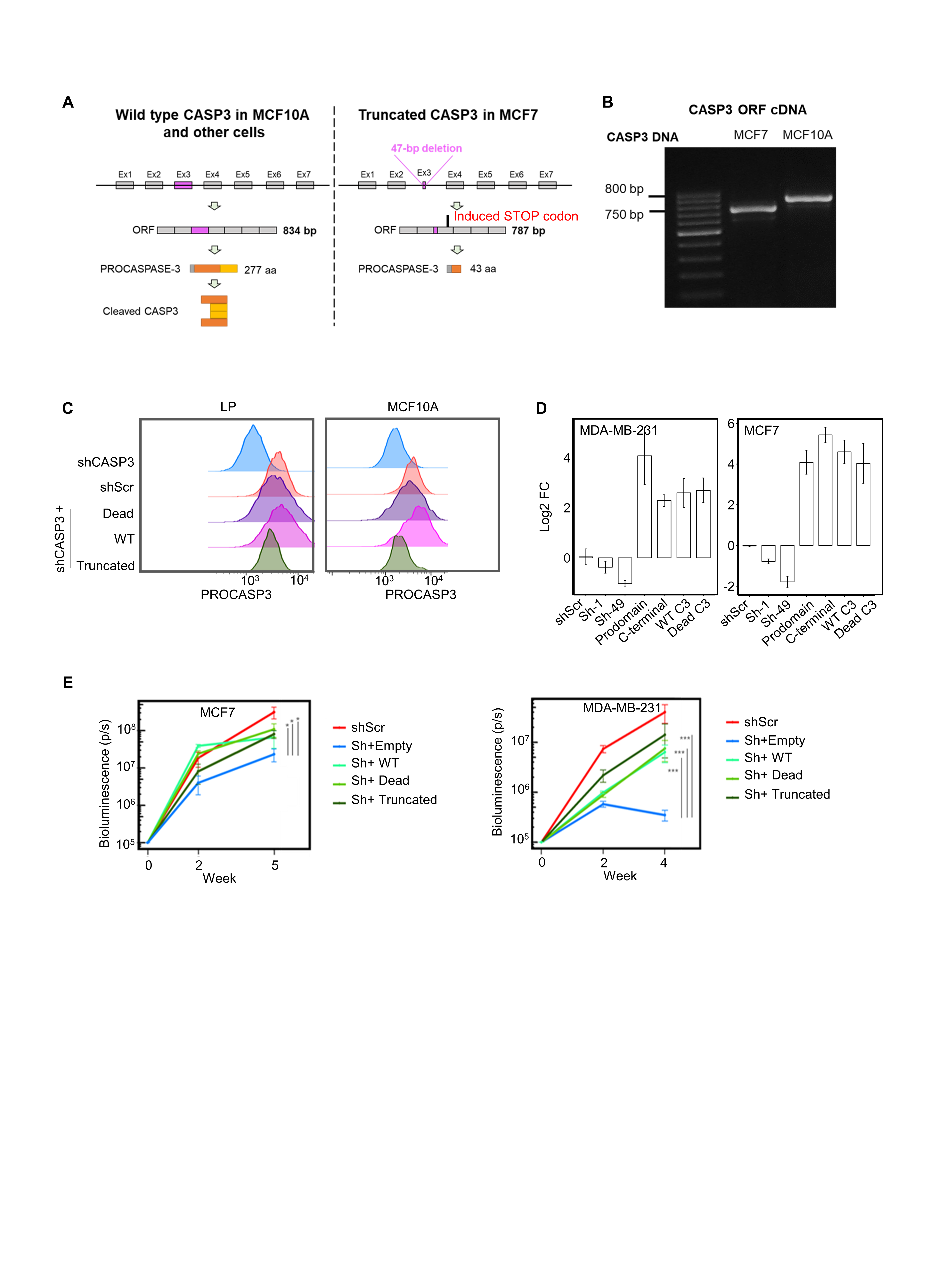

Supplement: Supplementary file 4 — S4 [file 41420_2024_1826_MOESM4_ESM.png]

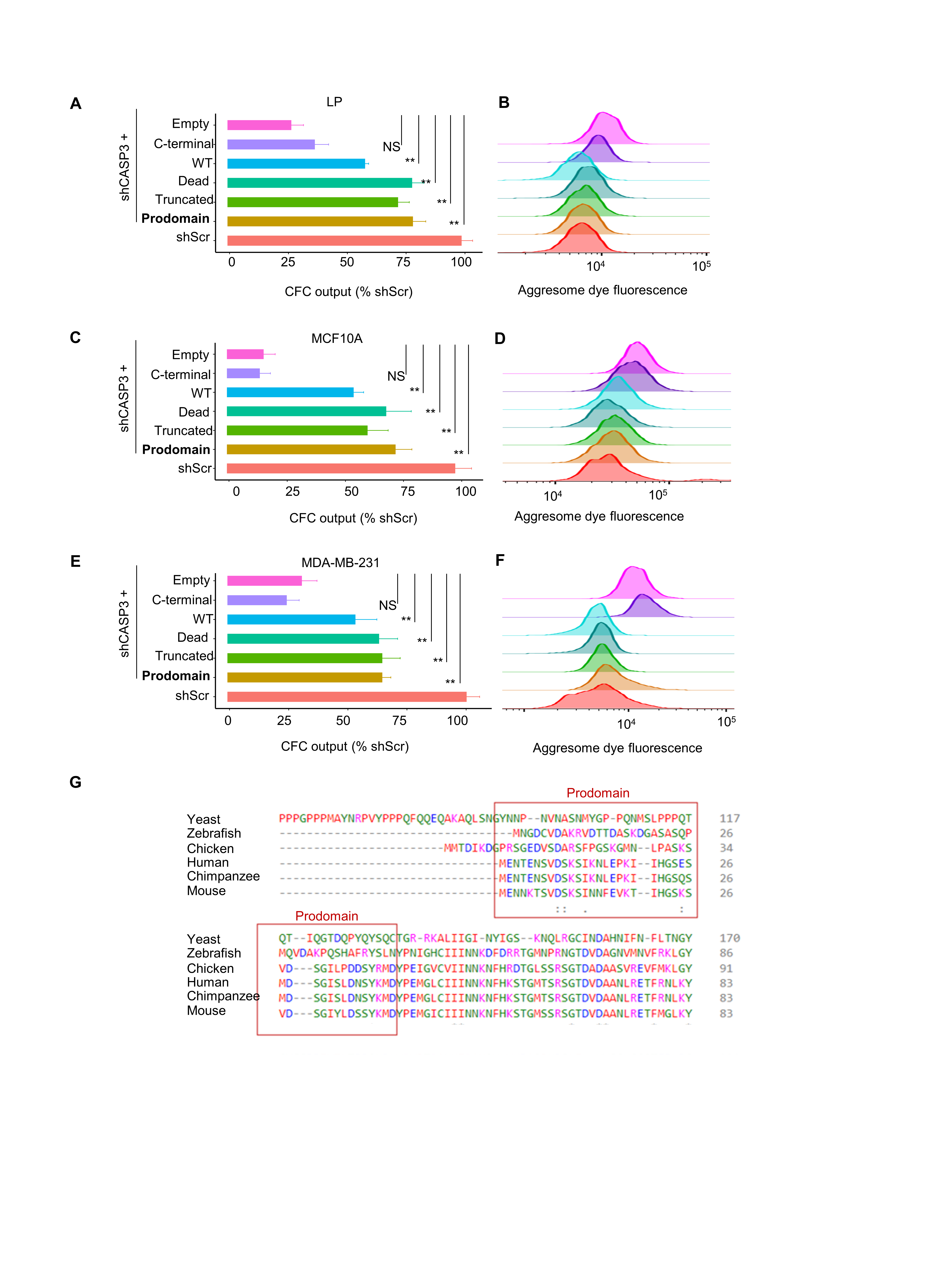

Supplement: Supplementary file 5 — S5 [file 41420_2024_1826_MOESM5_ESM.png]

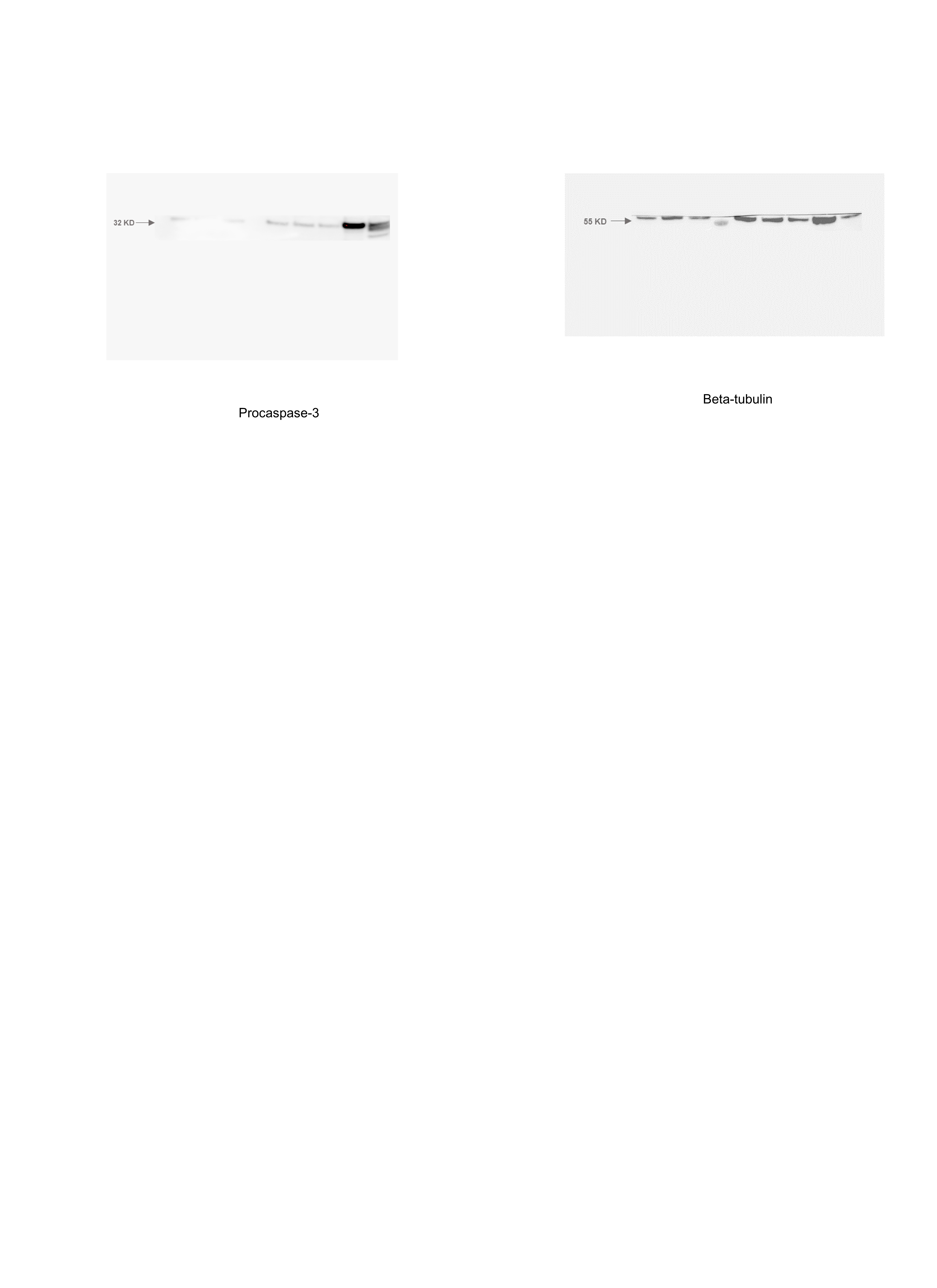

Supplement: Supplementary file 6 — Original Data File [file 41420_2024_1826_MOESM6_ESM.png]
