# Supplementary material for: Dependence of human cell survival and proliferation on the CASP3 prodomain
Source: Cell Death Discov. 2024 Feb 6;10:63. doi: 10.1038/s41420-024-01826-6 (PMC10847432; doi:10.1038/s41420-024-01826-6)
Supplement: Supplementary file 7 — Supplemental Material [file 41420_2024_1826_MOESM7_ESM.docx]

**Supplemental figure captions:**

**Supplemental Figure 1:** **shCASP3 cells show changes in their cell cycle profile** **A)** Flow cytometric detection of changes in PROCASPASE3 levels in LPs and MCF10A cells transduced with the shCASP3 or the shScr vector and examined after staining with an anti-PROCASPASE Ab. **B)** The total cell output/well in wells containing ≥2 cells 4 days after initiation of the single cell cultures (***P<0.001, Student’s t-test). **C)** Forced expression of shCASP3 does not alter the level of single and double-strand DNA breaks. LPs (a pool of cells from 5 donors) and MCF10A cells were exposed to shCASP3 or shScr vectors, transduced cells isolated by FACS 48 h later, cultured for 2 more days (10^4^ cells/cm^2^/group), and then harvested, fixed/permeabilized and stained with γ-H2AX and RPA2 Abs. **D)** Workflow used to generate and FACS analysis of clonal isolates of Fucci-transduced MCF10A, MDA-MB-231, and MCF7 cells for cell cycle tracking experiments. **E)** Time-course of cell cycle changes (% S-G2-M) in Fucci expressing cells over 8 days in shCASP3 as percentage of their corresponding shScr-transduced cells. **F)** Heatmap illustrating the quantified difference in Mean Fluorescent Intensity (MFI) obtained for multiple markers of cell proliferation and G0-G1 progression into S-phase. The colour bar indicates the fold change of the MFI for each marker in the shCASP3- compared to the shScr-transduced cells. Values <1 indicate downregulated proteins and those >1 represent upregulated proteins in the shCASP3-transduced cells. **G)** Inability of increased expression of cell cycle regulators to rescue the impaired growth of shCASP3-transduced cells *in vitro*. FACS selected MCF10A and MDA-MB-231 cells co-transduced with shCASP3 and vectors that encode cDNAs for CDK4, CDK6, and Cyclin D. were cultured at 50 cells/cm^2^ and the number of colonies present counted 7 days later. Shown are the mean ± SEM of 3 CFC assay replicates in the experiments performed with each cell line (***P<0.001, Student’s t-test).

**Supplemental Figure 2: The correlation among tested samples based on their global proteome profile** A) Workflow for the proteome profiling MS experiments. Freshly purified normal human mammary LPs and malignant MDA-MB-231 were exposed to shCASP3 or shScr vectors and those transduced (106 cells/group) were then isolated 3 and 5 days later used for MS proteome analysis. As a positive control for apoptotic cells, a similar aliquot of MDA-MB-231 cells were cultured for 3 days (104 cells/cm2) and treated with STS for 18 hours, washed, and harvested for the MS proteome analysis. B) The heatmap shows the Pearson correlation among tested samples based on their global proteome profile.

**Supplemental Figure 3: Changes in the protein levels related to autophagy and ER stress pathways** **A)** The cartoon shows the mechanism of action of the aggresome detection reagent (molecular rotor dye), and the workflow of the experiments undertaken to compare the levels of misfolded protein aggregates in shCASP3- or shScr-transduced cells. This method detects a molecular rotor dye that specifically intercalates into the cross-beta spine of quaternary protein structures present in misfolded and aggregated proteins, thereby blocking the rotation of the dye and its consequent emission of a strong fluorescent signal. **B)** Elevated levels of protein aggregates in MDA-MB-231 cells treated with 10μM proteasomal inhibitor (MG132) **C)** Time-course measurement of aggresome levels over 8 days in shCASP3 and shScr-transduced cells. **D)** Heatmaps showing the expression of proteins related to autophagy and the ER stress (unfolded protein response) in shCASP3 cells and shScr based on the MS proteomics data. The color code indicates Z-score values.

**Supplemental Figure 4:** **MCF7 cells reply on a truncated CASP3 for their proliferation** **A-B)** Validation of CASP3 gene deletion (47-bp) in MCF7 cells by PCR amplification of the CASP3 cDNA. **C)** Intracellular flow cytometry detection of PROCASPASE-3 levels in cells transduced with shCASP3 and CASP3 cDNA vectors. **D)** qPCR data showing the levels of Procaspase-3 in cells transduced with shCASP3 and CASP3 cDNA vectors. **E)** FACS-selected shCASP3- or shScr-transduced MCF7 and MDA-MB-231 cells co-transduced with the active, or inactive, or truncated inactive form and Luc were transplanted SQ into NRG mice (10^4^ cells, 3 mice/group) and tumour growth monitored by the subsequent levels of bioluminescence measured. Shown are the mean ± SEM data for 3 mice/group; (***P<0.001, Student’s t-test at week 4).

**Supplemental Figure 5. CASP3 prodomain regulates survival and aggregate levels in human mammary cells A, C, E)** Different CASP3 cDNAs were overexpressed for the rescue experiment on LPs, MCF10A, and MDA-MB-231. Shown are the mean ± SEM of 3 biological replicates performed on each of the 3 cell types tested. **B, D, F)** A representative series of histograms expressing all forms of CASP3 cDNAs tested and showing levels of protein aggregates in each group (from analyses of 10^5^ cells/group/cell type analyzed). **G)** Conservation of CASP3 prodomain among mammals.

**Tables**

**Table 1: Antibodies used for FACS isolation of human mammary subsets**

| **Antibody** | **Fluorophore** | **Clone** | **Company** |
| --- | --- | --- | --- |
| Mouse anti-human CD45 | Pacific Blue | HI30 | Biolegend |
| Mouse anti-human CD31 | Pacific Blue | WM59 | Biolegend |
| Mouse anti-human CD49f | APC | GoH3 | Biolegend |
| Mouse anti-human EpCAM | PE | 9C4 | Biolegend |
| Annexin V | FITC | 640906 | Biolegend |

**Table 2: Antibodies used for intracellular flow cytometry**

| **Antibody** | **Fluorophore** | **Clone** | **Company** |
| --- | --- | --- | --- |
| Autophagy Ab sampler kit (#4445) | NA | NA | CST |
| CDK2 | NA | 78B2 | CST |
| CDK6 | AF647 | EPR4515 | Abcam |
| CDK4 | NA | D9G3E | CST |
| Cyclin C | NA | E6V4Z | CST |
| Cyclin D | NA | E3P5S | CST |
| Isotype control IgG | NA | E5Y6Q | CST |
| P21 | NA | 12D1 | CST |
| pRb (pS807/pS811) | AF647 | J112-906 | BD |
| Secondary anti-rabbit (#8889) | AF594 | NA | CST |

| **shRNA** | **Sequence** |
| --- | --- |
| shCASP3-1 (targeting 3`-UTR) | AAGACATACTCCTTCCATCAA |
| shCASP3-2 (targeting 3`-UTR) | AAAGACCTATGAGCACATAGG |
| shCASP3-3 (targeting 3`-UTR) | AATGAGCTCGCATTTGTCAAT |
| Caspase3-49 (Targeting exon 4) | TTCCAGAGTCCATTGATTCGCT |
| Caspase3-50 (Targeting exon 4) | TATACATAAACCCATCTCAG |
| Caspase3-51 (Targeting exon 8) | ATTCTGTTGCCACCTTTCGG |
| shScr (Scramble control)-1 | GCCTTAAAGCCGTTATAGATT |
| shScr (Scramble control)-2 | TATTATTACGCTTATACGCGG |

**Table 3: shRNA Sequences used for CASP3 knockdown experiments**

**Table 4: TMT plex design**

| **Channel** | **Plex1** | **Plex2** |
| --- | --- | --- |
| 126 | PIS | PIS |
| 127N | 9 | 10 |
| 127C | 1 | 2 |
| 128N | 13 | 14 |
| 128C | 5 | 6 |
| 129N | 11 | 12 |
| 129C | 3 | 4 |
| 130N | 15 | 16 |
| 130C | 7 | 8 |
| 131N | 17 | 18 |
| 131C | SM+iD | SM+iD |

**Table 5: The Pro/Cleaved CASP3 peptides used for isobaric peptide doping (isoDoping).**

| **Peptides** |
| --- |
| IIHGSESMDSGISLDNSYK |
| GTELDCGIETD |
| SGISLDNSYK |
| SGVDDDMACHK |
| GTELDCGIETDSGVDDDMACHK |
| MENTENSVDSK |
| LFIIQAZR |
| GTELDZGIETD |
| LEFMHILTR |
| SGTDVDAANLR |
| MDYPEMGLZIIINNK |
| SLTGKPK |

**Methods**

**Generation of viable single-cell suspensions from human breast tissue samples**

Tissue from reduction mammoplasty surgeries were collected with informed consent from healthy premenopausal women, as approved by the University of British Columbia Research Ethics Board. Excess fat was first removed and the tissue was then dissociated in a 2-step as previously described (1). Briefly, the tissue was initially minced with scalpels and then dissociated for ~18 hours overnight at 37ºC in DMEM F12 media (1:1, STEMCELL Technologies) supplemented with 2% BSA (Gibco), 300 U/ml collagenase (Sigma) and 100 U/ml hyaluronidase (Sigma). An initial centrifugation at 80×g for 4 minutes used to obtain “A” pellets rich in small fragments of mammary tissue. These “A” pellets were then cryopreserved at -180ºC in FBS (STEMCELL Technologies) containing 6% DMSO. Prior to use, cryopreserved “A” pellets were thawed and rinsed with Hank’s Balanced Salt Solution supplemented with 2% FBS (HF) and the fragments further dissociated in a 2-step into single cells by incubating the fragments at 37ºC in 2.5 mg/ml trypsin with 1 mM EDTA (STEMCELL Technologies), 5 mg/ml Dispase (STEMCELL Technologies) and 100 µg/ml DnaseI (Sigma), with washing of the cells in HF between each step. The resulting cell suspensions were then filtered through a 40 µm mesh to obtain single-cell suspensions and populations of cells highly enriched in BCs, LPs, LCs and SCs isolated by FACS as described below.

**FACS isolation of BCs, LPs, LCs and their associated SCs**

Single-cell suspensions obtained from “A” pellets were depleted of hematopoietic and endothelial cells using antibodies to CD45 and CD31, respectively. Cells were also exposed to DAPI to eliminate dead (DAPI+) cells. Anti-EpCAM and anti-CD49f were used to isolate the BC and LP fractions free of mature luminal cells and stromal cells. BCs were FACS sorted according to their CD45-CD31-EpCAMloCD49f+ phenotype. LPs were isolated by FACS according to their CD45-CD31-EpCAMhiCD49f+ phenotype. FACS was performed using a FACS Aria III or Fusion cell sorter (BD Biosciences). Table 2.1 lists the fluorochrome-labelled antibodies used.

**Cell lines**

MDA-MB231, MCF-10A, MCF7, HeLa, and BT-20 cells were originally obtained from ATCC and routinely maintained as cultures of adherent cells. D425 cell line was a gift of Dr. Paul Sorenson's lab at the University of British Columbia, while the human embryonic fibroblast-derived iPSC line was a gift of Dr. Andras Nagy lab at University of Toronto (2). ALM5 cell line was a gift of Dr. Mark Minden, University of Toronto (3). We ensured that all cell lines tested were free from mycoplasma contamination, and this was achieved through routine mycoplasma testing using PCR-based methods. MCF10A cells were cultured in DMEM F12 + 5% horse serum (STEMCELL Technologies), 20 µg/ml EGF, 5 µg/ml hydrocortisone, 100 ng/ml cholera toxin, 10 μg/ml human insulin (all from Sigma) and 1 mg/ml streptomycin (Life Technologies). MDA-MB-231, BM fibroblasts and BT-20 cells were cultured in DMEM F12 + 10% FBS, 1 mg/ml streptomycin (Life Technologies), and MCF7 cells cultured in DMEM F12 + 10% FBS, 10 μg/ml human insulin (Sigma) and 1 mg/ml streptomycin (Life Technologies). AML5, and D425 cells were cultured in DMEM F12 + 20% FBS, 1 mg/ml streptomycin (Life Technologies), and IPSCs were cultured in mTeSR™1 (STEMCELL Technologies).

**Lentiviral vectors**

Most of the lentiviral vectors used in this study were based on the MNDU3-PGK-fluorochrome backbone (4). They included an MNDU3-PGK-mCherry-KRASG12D vector, variants of a MNDU3-PGK-YFP-CBR-Luc derivative and the Fucci vector (5). The MNDU3-PGK-mCherry backbone was used to express cDNAs for WT CASP3, catalytic inactive and truncated inactive CASP3 forms (6). For the Fucci vector, both the Cdt1-mKusabira Orange (mKO) and Geminin-mVenus were cloned into a single vector. All of the sh-vectors were created using the pLKO.1-puro vector modified by replacing puro with BFP. shRNAs targeting exon or 3`-UTR regions of *CASP3* gene as well as scrambled *CASP3* shRNA were cloned into pLKO.1-BFP for the KD experiments. Table 2.3 shows the sequences of shRNAs used in this study. All vectors were sequenced verified, in some cases concentrated by ultracentrifugation, frozen in small aliquots and titred before and after on Hela cells. The MNDU3-PGK-mCherry backbone was used to express cDNAs of different CASP3 forms used in the rescue experiments. The rescue vectors were validated by qPCR, WB and intracellular flow cytometry.

**Transduction procedure and isolation of transduced cells**

Human mammary cells were first exposed to one or more vectors in SF-7 media supplemented with 5% FBS (7), which creates a hybrid DMEM/F12 (STEMCELL Technologies) as the base medium supplemented with 0.1% BSA, 0.5 µg/ml hydrocortisone, 1 µg/ml insulin, 10 ng/ml EGF (Sigma) and 10 ng/ml cholera toxin (Sigma) (1). The cells obtained were then exposed to selected test and control vectors at ≤10^6^ cells in 100 µl of SF-7 media supplemented with 5% FBS 37°C and 20% O2 for 3 days on tissue culture petri dishes coated with collagen for primary human mammary epithelial cells, and without collagen for human mammary cell lines. Lentiviral transduction was performed in liquid suspension cultures containing cells at a concentration of ≤10^6^ cells in 100 µl of SF-7 media supplemented with 5% FBS. The cells were then washed twice with HF prior to being used for any subsequent experimental procedure. Due to the high level of heterogeneity in Fucci vector-transduced MCF10A, MCF7 and MDA-MB-231 cells, these were cloned directly by FACS 2-3 days after vector exposure and then following their expansion *in vitro* then transduced with shCASP3-BFP or shScr-BFP vectors and analyzed for mKO and mVenus expression by FACS. Non-mammary cell types were cultured for 2 days and then exposed to the lentiviral vectors overnight at a concentration of ≤10^6^ cells in 1000µl culture media. The cells were washed twice with HF prior to being used for any subsequent experimental procedure.

**Western blot (WB) analyses**

Frozen samples were resuspended in RIPA buffer (CST #9806) supplemented with 1 µl PIC, incubated on ice and centrifuged at 10,000 rpm for 10 minutes. In a 96-well plate, samples were resuspended in Bradford solution (BioRad) and incubated at room temperature for 5 minutes. Next, samples were mixed with loading dye (Invitrogen), vortexed and boiled at 95ºC for 5 minutes. Samples were loaded with DNA ladder (Thermofischer) and ran at 200V for 40 minutes. A Polyvinylidene fluoride or polyvinylidene difluoride (PVDF) membrane was activated by washing in methanol, double distilled water and stored in transfer solution containing methanol, double distilled water and transfer buffer. Gel and membrane were sandwiched, topped with transfer buffer and run at 100V for 1 hour. Proteins were visualized by Ponceau S staining. The membrane was blocked in 5% BSA for 30 minutes and incubated with primary anti-Caspase-3 antibody (Cell Signaling #9662) overnight in a cold room (4^o^C). The membrane was washed with Tris-buffered saline with 0.1% Tween® 20 Detergent (TBST) and incubated with a secondary antibody (mouse anti-rabbit, IgG-Horseradish peroxidase, Santa Cruz Biotechnology) for 1 hour. Following further washing with TBST, the membrane was visualized using a chemiluminescent camera.

**Mass spectrometry (MS) analyses**

Samples were lysed in 200 µL of lysis buffer containing 4 M guanidine hydrochloride (Sigma cat#G4505-500G), 10 mM tris(2-carboxyethyl) phosphine (Sigma, cat#C4706-2G) and 40 mM chloroacetamide (Sigma, cat#22790), 5 mM EDTA (Invitrogen, cat#AM9260G), 1x complete protease inhibitor cocktail, EDTA free (Sigma cat#4693132001), and 1x phosSTOP inhibitor (Sigma, cat#4906845001) in HPLC grade water (Sigma, cat#270733). Lysis was performed in a FastPrep24 bead beater (MP Biomedical) using Lysing D Matrix tubes (Cedarlane Laboratories, cat#116913100) at 6.0 M/s for 45 s twice with a 40 s rest in between cycles. Tubes were centrifuged at 20,000 xG for 1 min and the supernatant was recovered and heated for 15 minutes at 95 °C with 1200 rpm of mixing. The protein concentration was measured by BCA (Pierce, cat#23225) following the manufacturer’s instructions and 100 µg of protein was diluted to 500 µL in 20 mM HEPES (Sigma, cat#H3784-100G) pH 8 and 2 µg of sequencing grade trypsin/LysC (Promega, cat#V5071) was added. Protein was digested overnight at 37 °C with 1200 rpm mixing. Cell lines were labeled with Tandem Mass Tags (TMT, Thermo Fisher Scientific, cat#A34808) by the addition of 100 µg of each label in 10 µL of HPLC acetonitrile (Supplemental Table 4). The reaction was incubated at room temperature for 30 minutes. Another 100 µg of each label in 10 µL of acetonitrile was added and incubated for 30 minutes then the reaction was quenched by the addition of 10 µL of 1 M guanidine in HPLC water. Samples were combined and salt removed by SepPak desalting on a C18 column (Waters, cat#wat054960). The cartridge was rinsed twice with 1 mL of 0.1% trifluoroacetic acid in acetonitrile, twice with 0.1% trifluoroacetic acid in water, then sample was acidified with 1% trifluoroacetic acid and loaded onto the cartridge. Impurities were rinsed away with three aliquots of 1 mL of 0.1% formic acid in water then the peptides were eluted using two aliquots of 600 µL 0.1% formic acid in 80/20 HPLC grade acetonitrile/HPLC grade water. Volume was reduced to 200 µL in a vacuum centrifuge and subjected to offline fractionation on an Agilent prep fractionation system with a Kinetix EVO C18 column (2.1 x 150mm, 1.7 μm core shell, 100Å, Phenomenex). Elution was performed at a flow rate of 0.25 mL/min using a gradient of mobile phase A (10mM ammonium bicarbonate, pH 8) and B (acetonitrile), from 3% to 35% over 60 minutes. Fractions were collected every minute across the elution window for a total of 48 fractions, which were concatenated to 12 final fractions (e.g., 1 + 13 + 25 + 37 = fraction 1). Fractions were dried in a SpeedVac centrifuge and reconstituted in 0.1% formic acid in HPLC grade water for MS analysis.

The global proteome profiling was performed on an Thermo Orbitrap Eclipse MS coupled to a Thermo nLC 1200. Peptides were trapped on a fritted 100 µm ID column packed with 0.5 cm Dr. Maisch C18, 3 µm with 10 uL at 400 bar. Peptides were separated on a self-pulled column packed with 25 cm Dr. Maisch C18, 3 µm at a flow rate of 450 nL/min with solvent A as 0.1% formic acid in HPLC water and B as 0.1% formic acid in acetonitrile. The percent B was increased from 6% to 15% over 2 min, to 35% over 94 min, to 53% over 17 min. Solvent B was ramped to 100% over 1 min and the column was washed for 6 min, with a total gradient time of 120 min. Ions were formed using a voltage of 2400 V and a 3 s cycle of positive profile MS1 with a resolution of 120000 in the normal mass range using quadrupolar isolation over a scan range of 400-1200, standard AGC and auto max injection time. MIPS was set to peptide with a minimum and maximum intensity of 5.0e3 and 1.0e20, respectively. Charge states 2-6 and undetermined charge states were included for ddMS2 by HCD with a 0.7 *m/z* isolation window, 40% collision energy in the ion trap with a resolution of 50000 and a first mass of 120 *m/z*. To enable simultaneous measurement of pro and cleaved CASP3 levels, isobaric peptide doping (isoDoping) was used, Supplemental Table 5 (8). MS/MS were searched against the UniProt reference proteome (24635 sequences, 2020/02/18) using Sequest HT algorithm through the Proteome Discoverer suite (v2.4, Thermo Scientific). Precursor and fragment ion tolerance were set to 20 ppm and 0.05 Da, respectively. Minimum peptide length was set to 6 AA with a maximum of 2 missed cleavages allowed. Full Trypsin specificity was required. Dynamic modifications included Oxidation (+15.995 Da, M), Acetylation (+42.011 Da, N-Term), and static modification included Carbamidomethyl (+57.021 Da, C) and TMT (+229.163 Da, K, N-Term). Peptide-to-spectrum matches (PSMs) were calculated using Percolator by searching the results against a decoy sequence set; only PSMs with FDR< 1% were retained in the analysis.

Differentially expressed proteins between sample groups were calculated by the Differential Expression analysis of quantitative Mass Spectrometry data (DEqMS) R package (9). GSEA analysis was done using the tool from Broad institute (10) and pathways with q-value <0.15 were shown in the tables and heatmaps. Median t-statistics of the assigned peptides were used to calculate false-discovery rate-adjusted p-values determined from the beta distribution, as described previously (11).

**Single-cell tracking of survival and proliferation**

Clonal tracking of MCF10A and MDA-MB-231 (shCASP3- and shScr-transduced) cells was performed on single BFP+ sorted cells into separate wells of a 96-well plate containing 100μl of their respective filtered media. Following sorting of single-cells, each well was visually assessed to determine the presence or absence of a viable single highly refractile cell. Each well was then manually assessed 1x daily for 6 days for cell viability (high refractility, cell size, and membrane integrity) and cell division. Wells that were never observed to have a viable single cell in them through the course of the experiment were excluded from downstream analyses.

**Apoptosis assays**

Cultured cells were harvested and stained with Annexin V-FITC (Cat: 640906, BioLegend) and 1mg/ml PI (Sigma). They were then analyzed by FACS to distinguish different stages of apoptosis and death according to their staining with Annexin V only, both Annexin V and PI or PI only.

**Cell cycle analyses**

Cells were washed with cold PBS and stained with an eFluor™ 780 fixable viability dye (Invitrogen, #5016966) for 30 min on ice, then washed 1x in PBS + 20%. Cells were then fixed with 1.6% PFA for 10 min at room temperature, washed 1x with PBS + 2% FBS, then permeabilized by adding -80 °C 100% methyl alcohol (MeOH) dropwise while vortexing gently. Cells were then resuspended in a solution of 1 mg/ml PI, 10mg/ml DNase-free RNase in PBS and incubated at 37ºC for 30 minutes prior to FACS analysis.

**BrdU labeling**

Cultured cells were pulsed with 10 µM of BrdU (Biolegend) and incubated at 37°C for 2 hours. Cells were washed with cold PBS and stained with an eFluor™ 780 fixable viability dye (Invitrogen, #5016966) for 30 minutes on ice. Cells were washed, fixed with 95% ethanol, resuspended in 2N HCl and 0.1M NaB4O7 (pH 8.5) each for 30 minutes at room temperature (DNA hydrolysis step). Cells were then stained with Anti-BrdU antibody (APC-conjugated, Biolegend #339807), washed and then resuspended in a solution of 1 mg/ml PI and 10 mg/ml DNase-free RNase in PBS, and incubated at 37ºC for 30 minutes prior to FACS analysis of BrdU labeled cells.

**Aggrosome detection**

Abcam's Aggresome Detection kit (ab139486) (12–14) was used for quantification of aggrosome levels. Cultured cells were harvested, fixed with 4% Formaldehyde for 30 min at room temperature, and permeabilized with 0.5% Triton X-100, 3 mM EDTA, pH 8 for 30 minutes on ice. Then cells were resuspended in Aggresome Detection Reagent and incubated for 30 minutes, washed once with PBS and analyzed by flow cytometry. Positive control cells were pretreated with 5 µmol/L MG132 for 18 hours.

**Transplantation of mice**

Highly immunodeficient young adult virgin female NRG (NOD/Rag1-/-IL2rγc-/-) mice were used for all experiments according to procedures approved by the Animal Care Committee of the University of British Columbia. For generation of *de novo* tumours, freshly isolated BCs or LPs were transduced with *KRAS^G12D^* and Luciferase lentiviral vectors generated. Cells were transplanted SQ on the back of NRG mice, together with 50 µl of Matrigel in a total volume of 100 µl (Corning/BD Biosciences). For generation of tumors from cell lines, MDA-MB-231, MCF7, or BT-20 cell lines were exposed to shScr or shCASP3 and Luciferase vectors, and transduced cells mixed with 50 µl of Matrigel and implanted SQ into female NRG mice. Randomization and blinding was not utilized in the experimental design of the animal studies.

**Luciferase measurements**

Mice were injected intraperitoneally with 150 mg/kg body weight VivoGlo Luciferin (Promega) prepared in PBS and imaged 10 minutes later on a Xenogen IVIS Lumina In Vivo Imaging System with Living Image version 3.0 software (Caliper Life Sciences).

**Statistics**

Student’s t-test was used to compare the mean between two independent groups. Chi-squared test was used to compare frequency distributions in the cell cycle analyses. The null hypothesis was no difference in these proportions for shCASP3 and shScr control conditions. Analysis of survival and proliferation kinetics was performed in R. Survival was calculated using the functions ‘Surv’ and ‘survfit’, and statistical differences were calculated with a log-rank test using the ‘survdiff’ function in the ‘survival’ package.

**Reference:**

1. Eirew P, Stingl J, Raouf A, Turashvili G, Aparicio S, Emerman JT, et al. A method for quantifying normal human mammary epithelial stem cells with in vivo regenerative ability. Nat Med. 2008 Dec;14(12):1384–9.

2. Woltjen K, Michael IP, Mohseni P, Desai R, Mileikovsky M, Hämäläinen R, et al. piggyBac transposition reprograms fibroblasts to induced pluripotent stem cells. Nature. 2009 Apr 9;458(7239):766–70.

3. Wang C, Koistinen P, Yang GS, Williams DE, Lyman SD, Minden MD, et al. Mast cell growth factor, a ligand for the receptor encoded by c-kit, affects the growth in culture of the blast cells of acute myeloblastic leukemia. Leukemia. 1991 Jun;5(6):493–9.

4. Nguyen LV, Pellacani D, Lefort S, Kannan N, Osako T, Makarem M, et al. Barcoding reveals complex clonal dynamics of de novo transformed human mammary cells. Nature. 2015 Dec 10;528(7581):267–71.

5. Zielke N, Edgar BA. FUCCI sensors: powerful new tools for analysis of cell proliferation. Wiley Interdiscip Rev Dev Biol. 2015 Oct;4(5):469–87.

6. Stennicke HR, Salvesen GS. Biochemical characteristics of caspases-3, -6, -7, and -8. J Biol Chem. 1997 Oct 10;272(41):25719–23.

7. Raouf A, Zhao Y, To K, Stingl J, Delaney A, Barbara M, et al. Transcriptome analysis of the normal human mammary cell commitment and differentiation process. Cell Stem Cell. 2008 Jul 3;3(1):109–18.

8. Asleh K, Negri GL, Spencer Miko SE, Colborne S, Hughes CS, Wang XQ, et al. Proteomic analysis of archival breast cancer clinical specimens identifies biological subtypes with distinct survival outcomes. Nat Commun. 2022 Feb 16;13(1):896.

9. Zhu Y, Orre LM, Zhou Tran Y, Mermelekas G, Johansson HJ, Malyutina A, et al. DEqMS: A Method for Accurate Variance Estimation in Differential Protein Expression Analysis. Mol Cell Proteomics. 2020 Jun;19(6):1047–57.

10. Mootha VK, Lindgren CM, Eriksson KF, Subramanian A, Sihag S, Lehar J, et al. PGC-1α-responsive genes involved in oxidative phosphorylation are coordinately downregulated in human diabetes. Nat Genet. 2003 Jul;34(3):267–73.

11. Lefort S, El-Naggar A, Tan S, Colborne S, Negri GL, Pellacani D, et al. De novo and cell line models of human mammary cell transformation reveal an essential role for Yb-1 in multiple stages of human breast cancer. Cell Death Differ. 2022 Jan;29(1):54–64.

12. Kim YM, Yum MS, Heo SH, Kim T, Jin HK, Bae JS, et al. Pharmacologic properties of high-dose ambroxol in four patients with Gaucher disease and myoclonic epilepsy. J Med Genet. 2020 Feb;57(2):124–31.

13. Liu Y, Wang X, Coyne LP, Yang Y, Qi Y, Middleton FA, et al. Mitochondrial carrier protein overloading and misfolding induce aggresomes and proteostatic adaptations in the cytosol. Mol Biol Cell. 2019 May 15;30(11):1272–84.

14. Oe Y, Kakuda K, Yoshimura S ichiro, Hara N, Hasegawa J, Terawaki S, et al. PACSIN1 is indispensable for amphisome-lysosome fusion during basal autophagy and subsets of selective autophagy. PLoS Genet. 2022 Jun 30;18(6):e1010264.
